# Syntrophic Interaction between an Anoxygenic Photosynthetic Bacterium and a Tetrathionate-reducing Bacterium in Anaerobic Benzoate Degradation

**DOI:** 10.1264/jsme2.ME24105

**Published:** 2025-03-11

**Authors:** Miao He, Shin-ichi Nishitani, Shin Haruta

**Affiliations:** 1 Department of Biological Sciences, Tokyo Metropolitan University, 1–1 Minami-Osawa, Hachioji, Tokyo 192–0397, Japan

**Keywords:** microbe-microbe interspecies interaction, anaerobic aromatic compound degradation, anoxygenic photosynthetic bacteria, tetrathionate respiration, marine sediment

## Abstract

The present study exami­ned bacteria that anaerobically degrade the aromatic compound, benzoate, and obtained enrichment cultures from marine sediments under illumination. The enrichment culture contained anoxygenic photosynthetic bacteria and non-photosynthetic bacteria. The photosynthetic strain PS1, a purple sulfur bacterium in the genus *Marichromatium*, was unable to utilize benzoate; however, when combined with the non-photosynthetic bacterial isolate, *Marinobacterium* sp. strain BA1, the co-culture grew anaerobically on benzoate in the presence of thiosulfate or tetrathionate. Based on the metabolic profiles of the co-culture and axenic cultures, the following syntrophic interactions were proposed. Strain PS1 oxidizes thiosulfate as the electron source for photosynthesis to produce tetrathionate and relies on carbon dioxide produced through benzoate degradation by strain BA1. Strain BA1 oxidizes benzoate and reduces tetrathionate to provide thiosulfate to strain PS1 for photosynthetic carbon fixation. To the best of our knowledge, this is the first study to report anaerobic benzoate degradation in a photosynthetic co-culture through the syntrophic exchange of sulfur compounds.

Aromatic compounds, such as phenol and toluene, are widely distributed in nature as components of lignin and crude oils (Wilkes and Schwarzbauer, 2010; Lundquist and Parkås, 2011) and are prone to accumulation under anoxic conditions (Weelink *et al.*, 2010). The anaerobic biodegradation of aromatic compounds is critical for maintaining global material cycling and supporting environmental conservation (Weelink *et al.*, 2010).

Benzoate is a pivotal intermediate in the anaerobic biodegradation of a broad spectrum of aromatic compounds, making it a model compound for studying the degradation of these substances (Carmona *et al.*, 2009). Various microorganisms that degrade benzoate through anaerobic respiration processes, including nitrate, iron (III), and sulfate respiration, have been identified (Drzyzga *et al.*, 1993; Anders *et al.*, 1995; Coates *et al.*, 2001; Junghare and Schink, 2015; Wu *et al.*, 2015; Yang *et al.*, 2022). Additionally, the syntrophic utilization of benzoate, which involves cooperative interactions between fermentative bacteria and hydrogenotrophic methanogens, has been documented (Mountfort *et al.*, 1984; Schocke and Schink, 1997; Elshahed *et al.*, 2001; Qiu *et al.*, 2003).

Anoxygenic photosynthetic bacteria, known for their ability to utilize aromatic compounds, including benzoate, as carbon and electron sources represent another fascinating avenue for anaerobic degradation (Gibson and Harwood, 2002). Extensive studies have been conducted on photosynthetic benzoate degradation in soil and freshwater systems (Pfennig *et al.*, 1965; Pfennig, 1978; Wrightt and Madigan, 1991; Finneran *et al.*, 2003; Ramana *et al.*, 2006; Suresh *et al.*, 2020). However, to the best of our knowledge, their roles in marine environments remain unclear. Marine sediments, particularly in coastal zones, are critical sites for the accumulation of aromatic compounds, driven by both natural processes and anthropogenic activities (Arndt *et al.*, 2013; Cravo-Laureau and Duran, 2014). In the present study, we investigated anoxygenic photosynthetic bacteria from marine sediments that utilize benzoate and successfully obtained enrichment cultures that grew on benzoate under illumination. Two species of bacteria were isolated from the enrichment culture and their metabolic interactions were investigated.

## Materials and Methods

### Enrichment culture

Marine sediments (top 0–20‍ ‍cm) were collected from a tidal zone in Tokyo Bay (35.67N 139.78E) (Tokyo, Japan) and inoculated into a benzoate enrichment culture medium, in which benzoate served as the sole carbon source. The medium comprised the following (L^–1^): 2.0‍ ‍g sodium benzoate, 0.5‍ ‍g Na_2_S_2_O_3_·5H_2_O, 0.5‍ ‍g NH_4_Cl, 30‍ ‍g NaCl, 0.05‍ ‍g KH_2_PO_4_, 0.28‍ ‍g K_2_HPO_4_, 5‍ ‍mL of basal salt solution, and 1‍ ‍mL of vitamin mixture. The pH of the medium was adjusted to 7.5 using NaOH. Basal salt solution and the vitamin mixture were prepared as described by Hanada *et al.* (1995). Anaerobic conditions were achieved in a screw-capped glass test tube (ϕ18‍ ‍mm, volume of 32‍ ‍mL) filled with the medium. Sediment samples were cultivated at 30°C in the light (tungsten lamp with a 750-nm longpass filter), undergoing repetitive subcultivation. The absorption spectra (550–1,000‍ ‍nm) of the cultures were measured using a spectrophotometer (UV-1800; Shimadzu).

### Bacterial isolation

Bacteria were isolated using a single colony isolation method. Cultures were spread on 1.5% (w/v) Bacto-Agar plates containing the benzoate enrichment culture medium as described above or an alternative PO medium and incubated aerobically in the dark or anaerobically in the light. The PO medium contained the following (L^–1^, pH 7.5): 0.25‍ ‍g sodium formate, 0.25‍ ‍g sodium butyrate, 0.25‍ ‍g disodium succinate hexahydrate, 0.25‍ ‍g casamino acids, 0.5‍ ‍g Na_2_S_2_O_3_·5H_2_O, 0.5‍ ‍g (NH_4_)_2_SO_4_, 30‍ ‍g NaCl, 0.05‍ ‍g KH_2_PO_4_, 0.28‍ ‍g K_2_HPO_4_, 5‍ ‍mL of the basal salt solution, and 1‍ ‍mL of the vitamin mixture. Anaerobic conditions were established using an oxygen absorber (Ever-Fresh; Torishige Sangyo).

### 16S rRNA gene sequence ana­lysis

DNA extraction was performed as previously described by Noll *et al.* (2005). The 16S rRNA gene fragment was PCR-amplified using the 27F and 907R primers (Muyzer *et al.*, 1995; Suzuki and Giovannoni, 1996), and amplified DNA was sequenced using BigDye terminator kit v3.1 on an ABI3130 Genetic Analyzer (Applied Biosystems) (Otaki *et al.*, 2012). Sequences were compared against the rRNA/ITS databases from GenBank using BLAST under default settings at the National Center for Biotechnology Information (Camacho *et al.*, 2023).

### Genome ana­lysis

DNA was extracted from bacterial cells using a Qiagen Genomic-tip 100/G (Qiagen) and sequenced by Bioengineering Lab. g-TUBE (Covaris) was used to shear DNA (target length, approximately 10–15‍ ‍kb), followed by the construction of a sequencing library using the SMRTbell gDNA Sample Amplification Kit (PacBio) and SMRTbell Express Template Prep Kit (PacBio). A library polymerase complex was formed using the Revio Polymerase Kit (PacBio) and sequenced using Revio (PacBio). SMRT Link v13.0.0.207600 (PacBio) was used to obtain HiFi reads. PCR adapters, duplicates, and ≤1,000-bp short reads were removed using lima (ver. 2.7.1), pbmarkdup (ver.1.0.3), and Filtlong (ver. 0.2.1), respectively. Filtered HiFi reads were assembled using Flye (ver. 2.9.2-b1786). The completeness of assembled genome data was confirmed using CheckM2 (ver.1.0.1). Genomes were annotated using the DFAST pipeline (Tanizawa *et al.*, 2018). Default settings were used for all software ana­lyses.

### Co-cultivation of two species of bacteria

Bacterial isolates were pre-cultured aerobically in a benzoate-thiosulfate medium (see below) or photo-autotrophically in an inorganic medium (PI medium). The PI medium was prepared by removing benzoate from the benzoate enrichment medium and adding 3.0‍ ‍g‍ ‍L^–1^ of NaHCO_3_ in 32-mL glass test tubes sealed with butyl rubber stoppers and screw caps after replacing the gas phase with N_2_:CO_2_ (4:1). Bacterial growth was monitored by measuring optical density (OD) at 660‍ ‍nm (miniphoto 518R; Taitec). One milliliter of each culture at the stationary phase of the growth was inoculated into 32-mL test tubes filled with the benzoate-thiosulfate medium containing the following (L^–1^, pH 7.5): 0.5‍ ‍g sodium benzoate, 0.5‍ ‍g Na_2_S_2_O_3_·5H_2_O, 0.5‍ ‍g NH_4_Cl, 30‍ ‍g NaCl, 0.05‍ ‍g KH_2_PO_4_, 0.28‍ ‍g K_2_HPO_4_, 5‍ ‍mL of the basal salt solution, and 1‍ ‍mL of the vitamin mixture. The tubes were cultivated at 30°C in the‍ ‍light (tungsten lamp with a 750-nm longpass filter). When indicated, the benzoate-tetrathionate medium was prepared by removing sodium thiosulfate and adding sodium tetrathionate dihydrate (0.06‍ ‍g‍ ‍L^–1^) to the benzoate-thiosulfate medium.

### Quantification of benzoate and sulfur compounds

Benzoate concentrations were quantified using HPLC (Chromaster; Hitachi High-Tech) equipped with a Triart-C18 column (YMC) and a UV detector at 215‍ ‍nm. The mobile phase contained methanol and 20‍ ‍mM H_3_PO_4_ (1:1) and flowed at 0.4‍ ‍mL‍ ‍min^–1^ at 40°C. Thiosulfate and tetrathionate concentrations were measured by ion-pair chromatography (Miura *et al.*, 1997) using the same HPLC system and column as described above. The mobile phase comprised 6‍ ‍mM tetrapropylammonium, 24.4‍ ‍mM acetic acid, and 20% acetonitrile. Samples were analyzed at 30°C with a flow rate of 0.5‍ ‍mL‍ ‍min^–1^ and chromatograms were measured at 230‍ ‍nm. Sulfate concentrations were colorimetrically assessed using the QuantiChrom Sulfate Assay Kit (BioAssay Systems) according to the manufacturer’s instructions using a spectrophotometer (Infinite 200 PRO; Tecan). To detect S^0^, the culture solution was centrifuged at 12,500×*g* for 2‍ ‍min to collect the precipitate. The precipitate was then dissolved in methanol and analyzed using a Hitachi HPLC system equipped with a LiChrosher 100 RP18 column (Merck) and L-4000 UV detector (265‍ ‍nm, Hitachi). Analysis conditions were as follows: mobile phase, methanol:water (20:1); flow rate, 0.5‍ ‍mL‍ ‍min^–1^; column temperature, 35°C (Stal *et al.*, 1984; Rethmeier *et al.*, 1997). Sulfur Sublimed (Fujifilm-Wako) was used as the standard for S^0^.

### Quantification of the total cellular protein content

Bacterial cells were collected from the culture solution by centrifugation at 8,000×*g* for 5‍ ‍min. Precipitates were incubated in 0.2 M NaOH and 2% sodium dodecyl sulfate solution at 95°C for 15‍ ‍min (Kawai *et al.*, 2019b). Protein concentrations were measured using a DC protein assay kit (Bio-Rad) and an Infinite 200 PRO spectrophotometer (Tecan) using bovine serum albumin as the standard.

### Quantitative PCR ana­lysis

DNA was extracted from the co-culture using a DNeasy Microbial kit (Qiagen). The PCR primer sets Mn_F (5′-GCTTGCTGTGACGTTAACA-3′) and Mn_R (5′-CTCTACCGTCATCTAGCTCA-3′) and Mc_F (5′-CATGGGTCTTGACGTTACTC-3′) and Mc-R (5′-CTCTATCAAACTCTAGCCAG-3′) were designed to specifically amplify the 16S rRNA gene fragment of bacterial strains in the genera *Marinobacterium* and *Marichromatium*, respectively. Specific amplification using each primer set was confirmed by DNA sequence ana­lyses of the PCR fragments obtained using a mixture of both genomic DNAs, and no interference from non-targeted DNA was also confirmed to quantitatively amplify target DNA. The StepOne Real-time PCR system (Applied Biosystems) was used with FastStart Universal SYBR Green Master Mix (Roche). The reaction mixture contained 10‍ ‍μL FastStart SYBR Green Master, 0.12‍ ‍μL of 50‍ ‍μM primers, 8.76‍ ‍μL water, and 1‍ ‍μL DNA solution. Real-time PCR was performed as follows: initial denaturation at 95°C for 10‍ ‍min followed by 40 cycles at 95°C for 15‍ ‍s and at 60°C for 1‍ ‍min. Fluorescence was detected at the end of each extension reaction. Genome copy numbers were calculated based on a standard curve using purified genomic DNA (0.001 to 10‍ ‍ng μL^–1^) from each strain. The standard genomic DNA concentration was spectrophotometrically measured using BioSpec-nano (Shimadzu).

### Nucleotide sequence accession number

16S rRNA gene sequences were deposited in the DDBJ/EMBL/GenBank databases with the accession numbers LC800302 and LC800303. The accession numbers of the genomic sequences were GCA_041155135 and GCA_041155115.

## Results

### Enrichment cultures in the benzoate medium

**Fig. 1.**
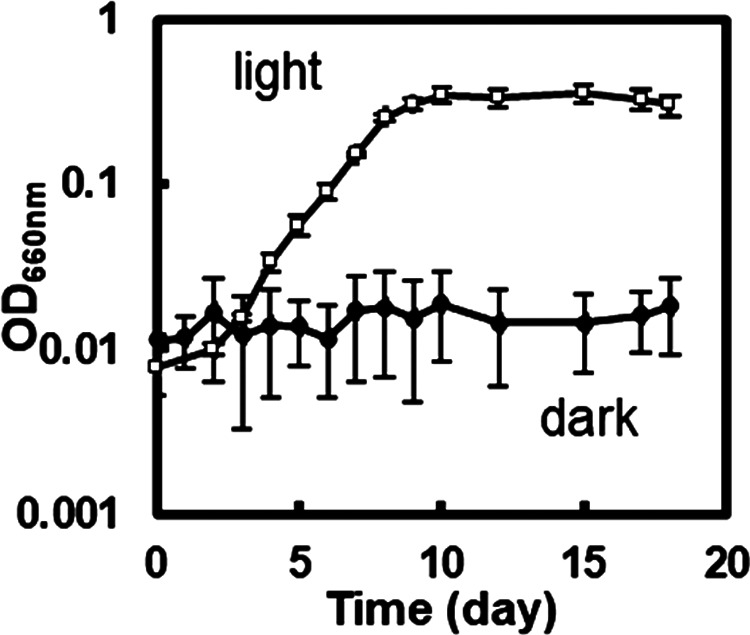
Growth curve of the benzoate enrichment culture from marine sediment. The enrichment culture was anaerobically cultivated in light (tungsten lamp with a 750-nm longpass filter) and dark. The optical density (OD) of the culture was measured at 660‍ ‍nm. Data are shown as the mean of triplicate culture tubes, and error bars show the standard deviation.

Approximately 1‍ ‍g of the sediments collected at Tokyo Bay were inoculated into 32‍ ‍mL of the benzoate enrichment culture medium in glass test tubes and cultivated at 30°C under oxygen-limiting conditions in the light. After 4‍ ‍weeks of cultivation, all 30 tubes showed a pinkish to reddish color, and a spectrophotometric ana­lysis of the cultures detected absorption peaks at approximately 800–900‍ ‍nm, indicating the growth of bacteriochlo­rophyll-containing bacteria (data not shown). After eight sub-cultivations, bacterial growth was repeatedly observed in five tubes and a representative is shown in Fig. 1. The OD value increased and reached a stationary phase after 9 days of cultivation. No increase in OD was detected in the dark (Fig. 1). All five cultures showed similar absorption spectra, with peaks at approximately 800 and 850‍ ‍nm (Fig. S1).

### Growth incapability of bacterial isolates in the benzoate medium

Anaerobic cultivation in light was conducted using the benzoate enrichment culture medium solidified with 1.5% agar after the inoculation with the enrichment culture. Colonies that emerged on the agar plate exhibited a red-white mosaic, indicating the presence of mixed colonies. Attempts to isolate single colonies from these mixed populations under the same culture conditions were unsuccessful. A subsequent aerobic culture yielded discrete whitish colonies. Additionally, anaerobic light cultivation in PO agar medium, containing several short-chain fatty acids, formed reddish colonies. We successfully isolated two distinct strains using a single colony isolation procedure: a whitish colony-forming strain, BA1, and a reddish colony-forming strain, PS1. The 16S rRNA gene fragment (~800 bp) was PCR-amplified from these strains and sequenced. A BLAST search revealed that close relatives of strain BA1 and strain PS1 were *Marinobacterium maritimum* (NR_116301, 99% identity) and *Marichromatium gracile* (NR_116468, 100% identity), respectively.

**Fig. 2.**
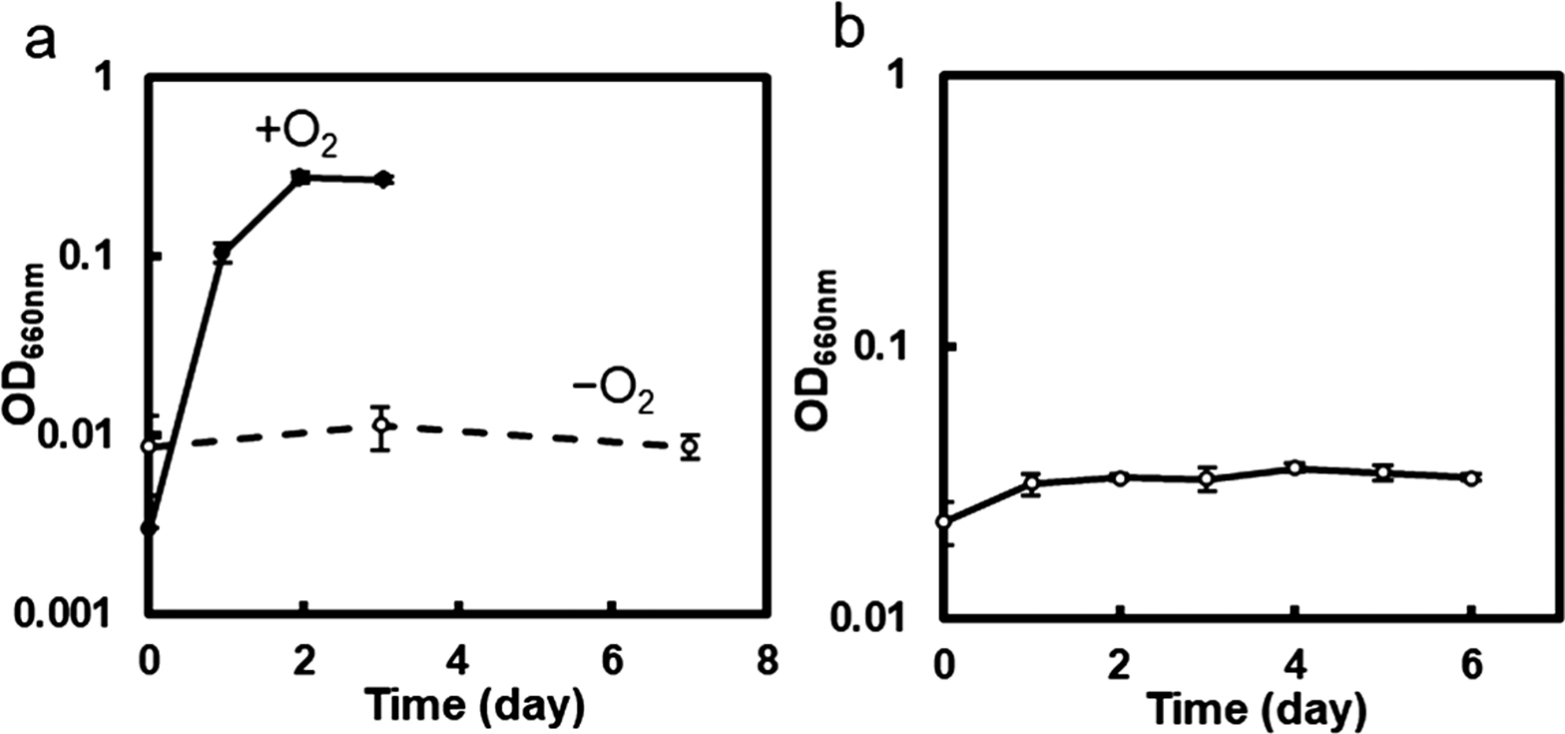
Growth curves of *Marinobacterium* sp. strain BA1 (**a**) and *Marichromatium* sp. strain PS1 (**b**) under axenic conditions. *Marinobacterium* sp. strain BA1 was cultivated in the benzoate-thiosulfate medium in the presence and absence of O_2_. *Marichromatium* sp. strain PS1 was anaerobically cultivated in the benzoate-thiosulfate medium. Anaerobic conditions were achieved by filling the culture tubes with the medium. The optical density (OD) of the culture was measured at 660‍ ‍nm. Data are shown as the mean of triplicate culture tubes, and error bars show the standard deviation.

Bacteria in the genus *Marinobacterium* in the class *Gammaproteobacteria* are chemoheterotrophic and aerobic/facultatively anaerobic (González and Buchan, 2021). *Marinobacterium* sp. strain BA1 grew in the benzoate-thiosulfate medium containing benzoate as the sole carbon source under aerobic conditions, as reported for *Marinobacterium* species (Park *et al.*, 2016), but did not grow in the absence of O_2_ (Fig. 2a). *Marichromatium* consists of marine purple sulfur bacteria in the class *Gammaproteobacteria* (Imhoff, 2015). *Marichromatium* sp. strain PS1 did not grow in the benzoate-thiosulfate medium under anaerobic light conditions (Fig. 2b); a slight increase in OD was supported by HCO_3_^–^, which was dissolved in the fresh medium and carried over from the pre-culture.

The genomic sequences of these isolates were determined (Table S1). *Marinobacterium* sp. strain BA1 possesses a comprehensive gene set for the aerobic benzoate-utilizing pathway, including a benzoate hydroxylase and dihydroxycyclohexadiene carboxylate dehydrogenase (Table S2) (Díaz *et al.*, 2013), but lacks genes for the dissimilatory reduction of sulfate, sulfur, iron (III), and nitrate. Genomic ana­lyses of *Marichromatium* sp. strain PS1 did not detect any homologous genes for benzoyl-CoA ligase, which is a crucial enzyme in anaerobic benzoate degradation (Harwood *et al.*, 1998), by a BLAST search with benzoyl-CoA ligase (WP_144359541) from *Sedimenticola selenatireducens* (GCF_000428045) (Narasingarao and Häggblom, 2006) in the class *Gammaproteobacteria* as a query. These genomic insights support that neither strain BA1 nor strain PS1 grew anaerobically in the benzoate-thiosulfate medium, as shown in Fig. 2.

### Co-cultivation on the benzoate medium containing thiosulfate

**Fig. 3.**
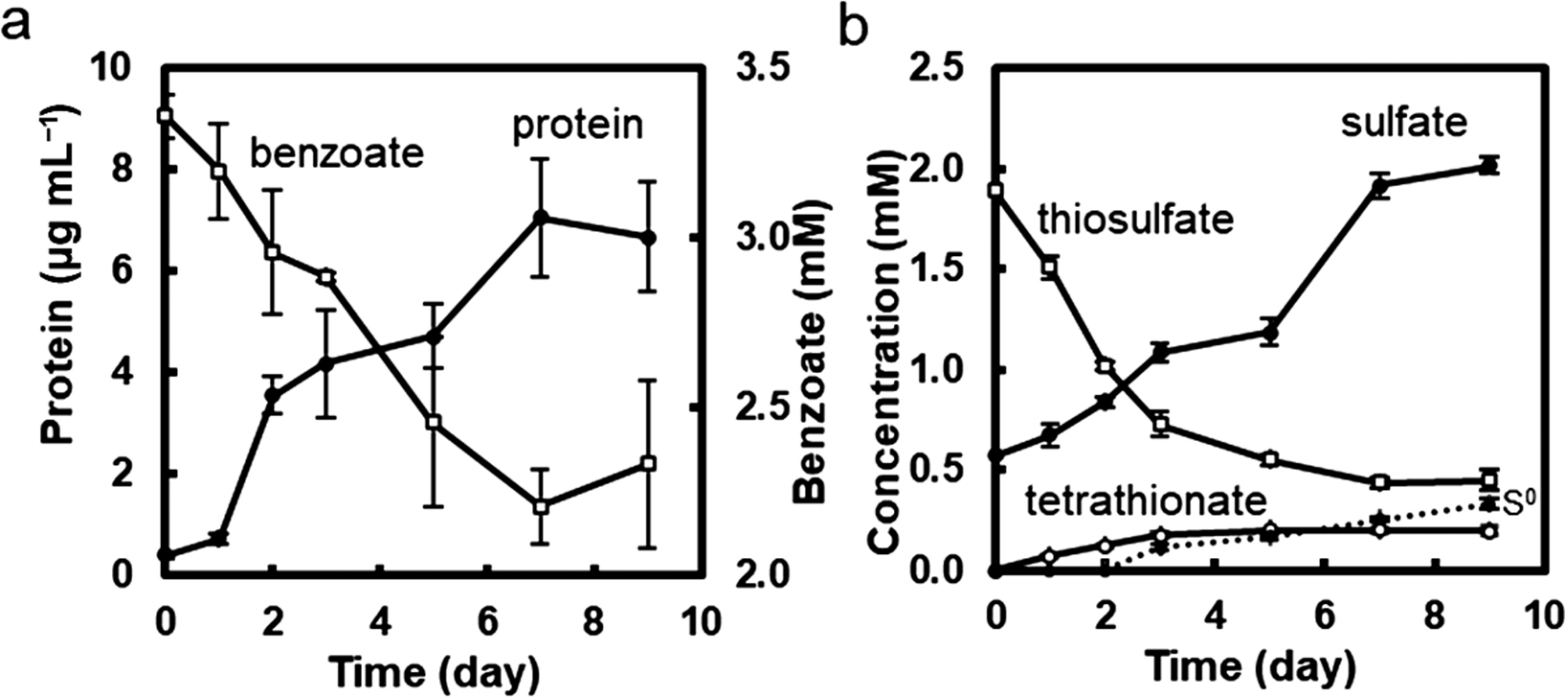
Co-cultivation in the benzoate-thiosulfate medium Strains BA1 and PS1 were co-inoculated, cultivated in the benzoate-thiosulfate medium under light, and repetitively sub-cultivated under the same conditions. Anaerobic conditions were achieved by filling the culture tubes with the medium. Data were obtained from the tenth sub-cultivation. **a**, Changes in total cellular protein and benzoate concentrations. **b**, Changes in the concentrations of sulfur compounds in the culture. Data at each time point are shown as the mean of three culture tubes, and error bars indicate the standard deviation.

We investigated the co-cultivation of *Marinobacterium* sp. strain BA1 and *Marichromatium* sp. strain PS1 in a medium containing benzoate as the sole carbon source and thiosulfate. Under oxygen-limiting conditions, growth was repetitively observed in light and the results of the tenth sub-cultivation are shown in Fig. 3. No growth was detected in the dark (Fig. S2). Growth was also not observed in a thiosulfate-free medium (Fig. S2), indicating that the effects of dissolved oxygen in the medium on growth were negligible. Total cellular protein content in the culture increased under light and became stable on day 7 (Fig. 3a). No change in the pH of the medium was confirmed after 7 days of cultivation. Total DNA was extracted on day 7 for quantitative PCR to examine the population ratios of the two strains. Genomic copy numbers mL^–1^ in the culture solution were estimated to be (4.00±0.52)×10^7^ for strain BA1 and (1.68±0.41)×10^7^ for strain PS1; this ratio was stable between the sub-cultures. Biomass production, as shown by an increase in cellular proteins, correlated with a decrease in benzoate concentrations (Fig. 3a). Concomitant with the increase in the cellular protein content and decrease in benzoate, a reduction was observed in thiosulfate levels from an initial concentration of 1.92±0.31‍ ‍mM to 0.45±0.05‍ ‍mM (Fig. 3b). Thiosulfate consumption was accompanied by an increase in sulfate concentrations. Tetrathionate was produced and its concentration increased until day 3 (Fig. 3b). S^0^ production was detected from day 3, and S^0^ levels continued to increase during the cultivation (Fig. 3b). S^0^ was microscopically observed as refractile globules in cells (Fig. S3). The accumulation of S^0^ in the culture may have prevented further growth and benzoate degradation (Libenson *et al.*, 1953).

### Possible factors enabling bacterial isolates to grow in the benzoate medium

**Fig. 4.**
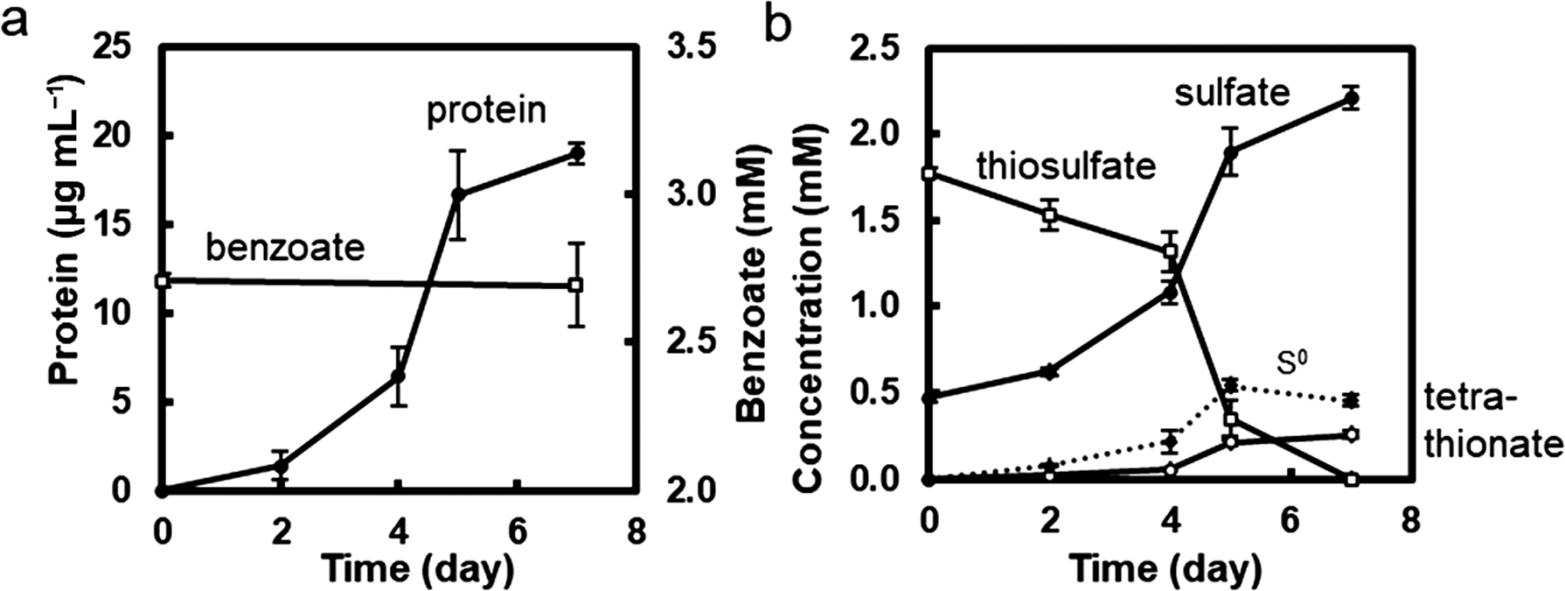
Anaerobic cultivation of *Marichromatium* sp. strain PS1 in the benzoate-thiosulfate medium supplemented with NaHCO_3_ Strain PS1 was cultivated in the benzoate-thiosulfate medium supplemented with NaHCO_3_ (0.03‍ ‍g mL^–1^) in the light. Anaerobic conditions were achieved by filling the culture tubes with the medium. **a**, Changes in total cellular protein and benzoate concentrations. **b**, Changes in the concentrations of sulfur compounds in the culture. Data at each time point are shown as the mean of three culture tubes, and error bars indicate the standard deviation.

Bacteria in the genus *Marichromatium* have been reported to grow photo-autotrophically using thiosulfate as an electron source (Imhoff, 2015). In the co-culture, *Marichromatium* sp. strain PS1 may have photosynthetically oxidized thiosulfate using CO_2_ as the carbon source. To confirm thiosulfate oxidation and the oxidized products, strain PS1 was axenically cultivated in the benzoate-thiosulfate medium supplemented with NaHCO_3_. Growth was confirmed by an increase in cellular proteins in the culture and benzoate consumption was not detected (Fig. 4a). Thiosulfate was gradually consumed and depleted on day 7. Concurrent with thiosulfate consumption, sulfate concentrations increased. Small amounts of S^0^ and tetrathionate were‍ ‍also produced (Fig. 4b and S3). As previously reported for a well-characterized purple sulfur bacterium in *Gammaproteobacteria*, *Allochromatium vinosum* (Hensen *et al.*, 2006), sulfate and tetrathionate may be the end products of thiosulfate oxidation, and S^0^ was an intermediate in the oxidation process to sulfate. These metabolic pathways were encountered in the genome of strain PS1 (Table S3). The consumption of thiosulfate, 3.6‍ ‍mM as S, was not recovered by the sum of the production of sulfate (1.7‍ ‍mM as S), S^0^ (0.5‍ ‍mM as S), and tetrathionate (1.1‍ ‍mM as S), *i.e*., 3.3‍ ‍mM in total. This may be due to the insufficient extraction efficiency of S^0^ from the culture, as suggested in previous studies (Prange *et al.*, 2002; Hensen *et al.*, 2006; Welte *et al.*, 2009).

**Fig. 5.**
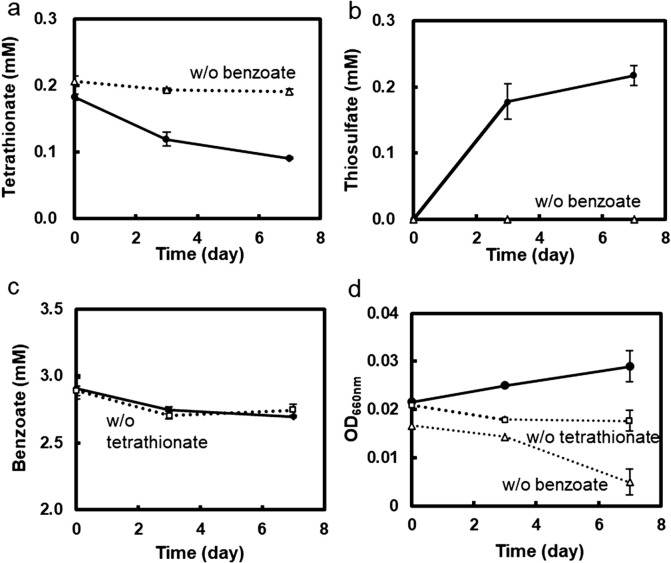
Anaerobic cultivation of *Marinobacterium* sp. strain BA1 with/without benzoate and tetrathionate Strain BA1 was cultivated in benzoate-tetrathionate (solid line with filled circles), benzoate-free (dotted line with open triangles), and tetrathionate-free media (dotted line with open squares). Anaerobic conditions were achieved by filling the culture tubes with the medium. **a**, Tetrathionate concentrations; **b**, thiosulfate concentrations; **c**, benzoate concentrations; **d**, optical density (OD) at 660‍ ‍nm. Data are shown as the mean of triplicate culture tubes, and error bars indicate the standard deviation.

We hypothesized that *Marinobacterium* sp. strain BA1 anaerobically reduced tetrathionate by utilizing benzoate as the electron donor in the co-culture. To experimentally test its ability for tetrathionate reduction and benzoate oxidation, strain BA1 was cultivated in the benzoate-tetrathionate medium under anaerobic conditions. Due to growth suppression by tetrathionate (Knox, 1945), a low concentration of tetrathionate, 0.2‍ ‍mM, was applied. Tetrathionate consumption and thiosulfate production were detected in the presence of benzoate (Fig. 5a and 5b), whereas benzoate consumption was not largely enhanced by approximately 0.1‍ ‍mM of tetrathionate reduction (Fig. 5c). Only a limited amount of benzoate (0.0067‍ ‍mM) was oxidized by the reduction of tetrathionate (0.1‍ ‍mM) to thiosulfate, as estimated by the chemical equation below.

C_7_H_5_O_2_^–^+15S_4_O_6_^2–^+19H_2_O → 7HCO_3_^–^+30S_2_O_3_^2–^+36H^+^

The increase in the OD value of the culture was detected in the presence of both benzoate and tetrathionate, while a deficiency in either benzoate or tetrathionate slightly decreased OD values (Fig. 5d). These results suggest the tetrathionate respiratory ability of *Marinobacterium* sp. strain BA1. The genome of *Marinobacterium* sp. strain BA1 contained a gene (MnBA_34150) homologous to the tetrathionate reductase gene (*tsdA*) of the tetrathionate respiratory bacterium *Campylobacter jejuni* (Liu *et al.*, 2013) (43.1% similarity in the deduced amino acid sequence) (Table S2). In the absence of tetrathionate, benzoate concentrations decreased (Fig. 5c), whereas the OD value did not increase (Fig. 5d). The incomplete oxidation of benzoate may occur; however, energy yields were not sufficient to maintain and produce the cellular biomass as reported for fermentative bacteria (Elshahed *et al.*, 2001).

### Co-cultivation on the benzoate medium containing tetrathionate instead of thiosulfate

**Fig. 6.**
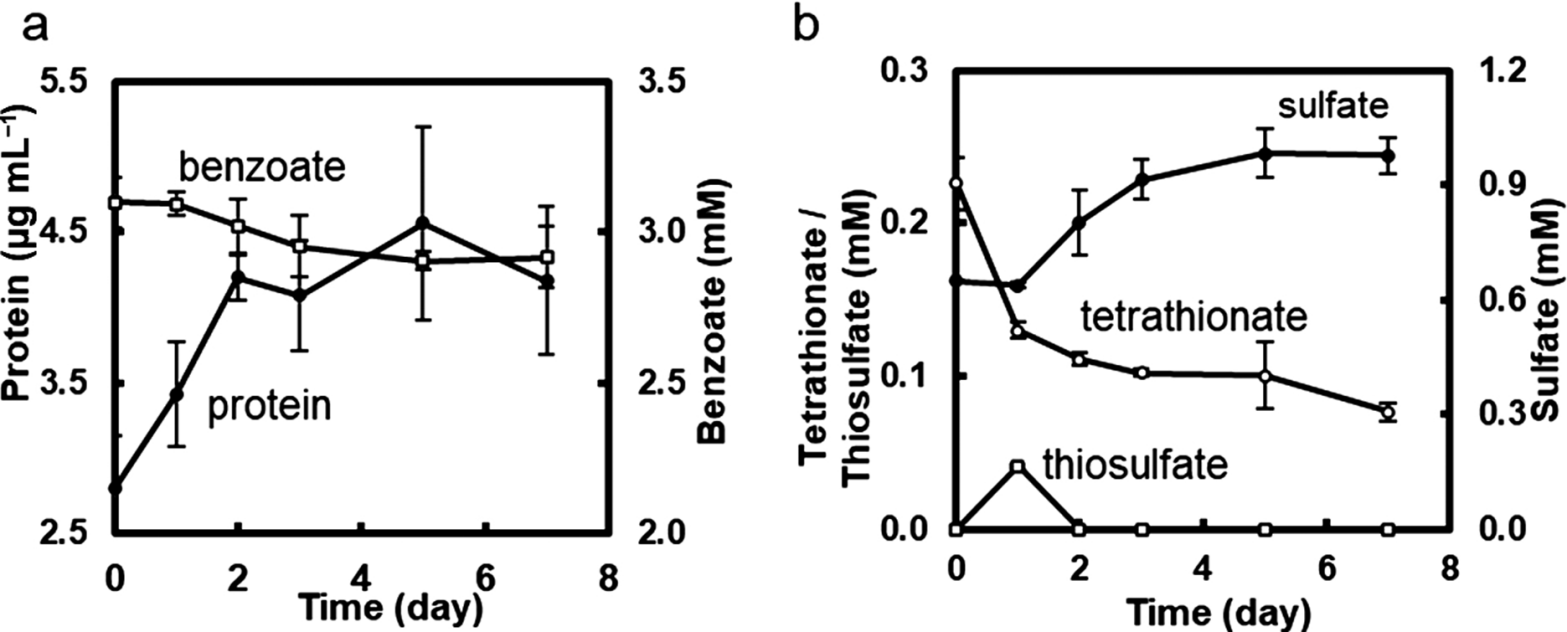
Co-cultivation in the benzoate-tetrathionate medium The co-culture solution of strains BA1 and PS1 in the benzoate-thiosulfate medium (as shown in Fig. 3) was inoculated and cultivated in the benzoate-tetrathionate medium in light. Anaerobic conditions were achieved by filling the culture tubes with the medium. **a**, Changes in total cellular protein and benzoate concentrations. **b**, Changes in the concentrations of sulfur compounds in the culture. S^0^ concentrations were below the detection limit (0.05‍ ‍mM) throughout the cultivation. Data at each time point are shown as the mean of three culture tubes, and error bars indicate the standard deviation.

The tetrathionate-reducing and benzoate-oxidizing abilities of *Marinobacterium* sp. strain BA1 (Fig. 5) suggest that‍ ‍strain BA1 provides oxidized products, thiosulfate, and possibly CO_2_ to the purple sulfur bacterium in the co-culture. To test this hypothesis, the co-culture was cultivated in the benzoate-tetrathionate medium (Fig. 6). An increase in cellular protein levels was observed, which was associated with decreases in benzoate (Fig. 6a) and tetrathionate concentrations (Fig. 6b). Thiosulfate was tentatively detected on day 1, and the sulfate concentration increased from days 1 to 5. The growth of *Marichromatium* sp. strain PS1 in the co-culture was confirmed by an increase in ab­sorb­ance at approximately 800 and 850‍ ‍nm, cor­re­sponding to bacteriochlo­rophylls (Fig. S4). The genomic copy numbers of two strains were similar: (2.53±0.88)×10^6^ copies mL^–1^ for strain BA1 and (1.62±0.57)×10^6^ copies mL^–1^ for strain PS1. These results suggest tetrathionate reduction by *Marinobacterium* sp. strain BA1 and thiosulfate oxidation by *Marichromatium* sp. strain PS1 in the co-culture.

## Discussion

**Fig. 7.**
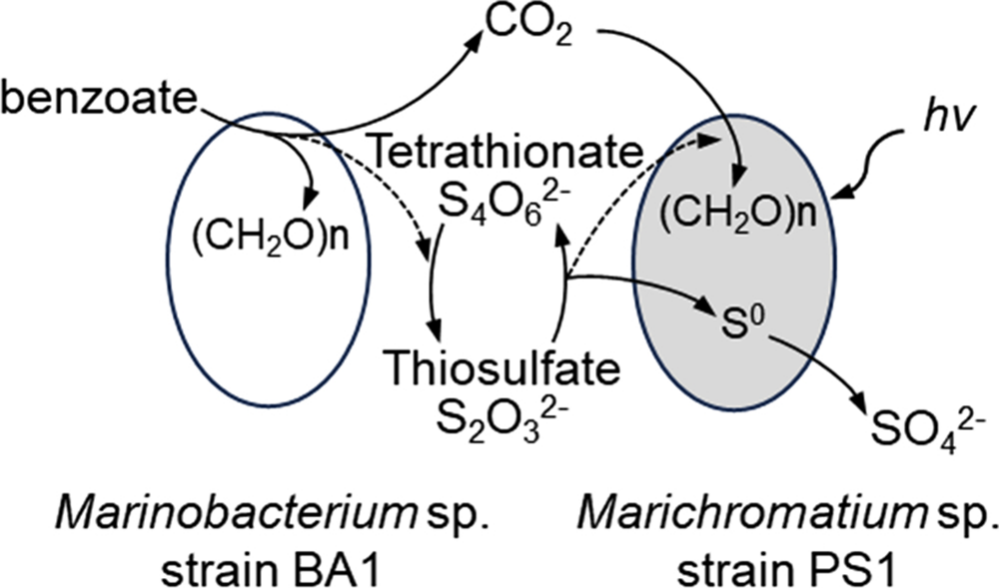
A schematic representation of syntrophic metabolic flows connecting benzoate oxidation (*Marinobacterium* sp. strain BA1) and photosynthetic thiosulfate oxidation (*Marichromatium* sp. strain PS1) Thiosulfate (S_2_O_3_^2–^) is oxidized by anoxygenic photosynthetic bacterium strain PS1 to produce tetrathionate (S_4_O_6_^2–^) in addition to sulfate (SO_4_^2–^) through S^0^. The tetrathionate provided by strain PS1 is reduced to thiosulfate by the benzoate-oxidizing bacterium strain BA1. Through benzoate oxidation by strain BA1, carbon dioxide is provided to strain PS1 as a carbon source. Dotted lines represent the flow of electrons.

In the present study, we exami­ned marine photosynthetic bacteria that anaerobically oxidize benzoate. Our enrichment culture, obtained from marine sediments, grew on benzoate under illumination (Fig. 1) and contained purple sulfur bacteria and non-photosynthetic bacteria. The photosynthetic isolate *Marichromatium* sp. strain PS1 did not oxidize benzoate (Fig. 2b and 4); however, a co-culture with the non-photosynthetic bacterial isolate *Marinobacterium* sp. strain BA1 resulted in anaerobic growth on benzoate under light conditions (Fig. 3). From the beginning of the co-cultivation, concomitant decreases were detected in benzoate and thiosulfate (Fig. 3), suggesting that the simultaneous growth of *Marinobacterium* sp. strain BA1 and *Marichromatium* sp. strain PS1 was due to their close metabolic interactions. This study proposes syntrophic interactions, as shown in Fig. 7. *Marichromatium* sp. strain PS1 oxidizes thiosulfate, as an electron source for photosynthesis, to produce tetrathionate and sulfate. *Marinobacterium* sp. strain BA1 oxidizes benzoate and reduces tetrathionate to provide carbon dioxide and thiosulfate to strain PS1 for photosynthesis.

In the axenic culture of *Marinobacterium* sp. strain BA1, tetrathionate was converted to thiosulfate in the presence of benzoate (Fig. 5). The genome of strain BA1 encodes a tetrathionate reductase (MnBA_34150), which may receive electrons via cytochrome *c* in the periplasmic space as part of the respiratory chain, as previously reported for dissimilatory tetrathionate-reducing bacteria (Liu *et al.*, 2013). To the best of our knowledge, this is the first study to report tetrathionate-reducing ability in the genus *Marinobacterium*. A possible tetrathionate reductase was widely found in various species of this genus: *Marinobacterium iners* (CFI10_RS16640, WP_206836632), *Marinobacterium marinum* (H1S06_RS10840, WP_181740040), *Marinobacterium sediminicola* (LN244_RS14425, WP_239040474), *Marinobacterium halophilum* (CLV44_RS16405, WP_106593205), *Marinobacterium littorale* (G403_RS0119415, WP_027855464), *Marinobacterium weihaiense* (KTN04_RS14085, WP_217335874), *Marinobacterium maritimum* (ABD013_RS06485, WP_343804035), *Marinobacterium stanieri* (BW956_RS20410, WP_076465941), and *Marinobacterium alkalitolerans* (H9C73_RS07670, WP_209287227). This result suggests a common potential for tetrathionate respiration in *Marinobacterium*.

Tetrathionate respiration was reported in 1923 for the enterobacterium, *Salmonella enterica* serotype *Typhimurium* (Muller, 1923). In the intestines, tetrathionate is produced by the chemical oxidation of thiosulfate by nitric oxide radicals and reactive oxygen species (Winter *et al.*, 2010). A genetic survey of the genes responsible for tetrathionate reduction suggested a wide distribution of tetrathionate respiratory bacteria in natural environments, including soil (Radl *et al.*, 2019), freshwater (Suhadolnik *et al.*, 2017; Vavourakis *et al.*, 2019), and marine environments (Flieder *et al.*, 2021). In these environments, tetrathionate may be produced via microbiological thiosulfate oxidation (Barrett and Clark, 1987). The genetic and metabolic responses of tetrathionate respiratory bacteria to their syntrophic partners warrant further study (van der Stel and Wösten, 2019).

The aerobic benzoate-oxidizing pathway found in *Marinobacterium* (Durán-Viseras *et al.*, 2021) is unlikely to work under anaerobic conditions because aerobic oxidation requires mole­cular oxygen (Fuchs, 2008). Anaerobic benzoate-oxidizing pathways reported in bacteria are initiated with benzoyl-CoA ligase (EC:6.2.1.25) followed by reductive dearomatization of the benzene ring (Carmona *et al.*, 2009). A BLAST search for these enzymes in *Marinobacterium* sp. strain BA1 was conducted to identify homologous genes from the anaerobic benzoate-degrading bacteria, *S. selenatireducens* in *Gammaproteobacteria*, *Thauera aromatica* in *Betaproteobacteria*, and *Geobacter metallireducens* in *Deltaproteobacteria* (Philipp and Schink, 2000; Wischgoll *et al.*, 2005; Carlström *et al.*, 2015). A protein sequence encoded by MnBA_13260 of *Marinobacterium* sp. strain BA1 showed 42–44% similarity with benzoyl-CoA ligase from these bacteria (Table S2), but no homologous gene for their benzoyl-CoA reductase was detected from strain BA1. Another type of dearomatizing enzyme may allow for benzoate degradation in *Marinobacterium* sp. strain BA1 under anaerobic conditions.

*Marichromatium* sp. strain PS1 oxidized thiosulfate to tetrathionate and sulfate (Fig. 4). Tetrathionate-producing purple sulfur bacteria are ubiquitous in nature and are found in areas such as marine sediments, soil, and freshwater environments (Imhoff and Trüper, 1980; Arunasri *et al.*, 2005; Shabeb *et al.*, 2008; Serrano *et al.*, 2009, 2015; Petushkova *et al.*, 2024). The expression of thiosulfate-oxidizing pathways to tetrathionate and sulfate encoded by *tsdBA*, *soxA*, *soxB*, *soxX*, *soxY*, and *soxZ* was not largely affected by growth conditions in a well-studied purple sulfur bacterium, *A. vinosum* (Weissgerber *et al.*, 2014). Notably, thiosulfate oxidation to sulfate yields more energy than its conversion to tetrathionate (Thauer *et al.*, 1977; Hensen *et al.*, 2006; Dahl, 2020), and the accumulation of tetrathionate is toxic (Knox, 1945; Parker and Alleon, 1969; Murphy *et al.*, 1972). The physiological advantage of this inefficient pathway for tetrathionate production is still being debated (Jørgensen *et al.*, 2019); however, syntrophic co-evolution with tetrathionate-reducing bacteria implies a possible advantage. The standard reduction potential (*E*^0’^) of the thiosulfate/tetrathionate couple is +0.025 V, which is higher than those of thiosulfate/sulfate (–0.245 V) and sulfide/sulfate (–0.215 V) (Brune, 1995), indicating that tetrathionate is an effective oxidizing agent. Therefore, photosynthetic tetrathionate production coupled with the anaerobic oxidation of aromatic compounds through tetrathionate reduction is preferable for microbial metabolism.

How is benzoate degraded in marine sediments? At the surface of sediments, where oxygen is abundant, benzoate undergoes aerobic degradation by aerobic bacteria, such as *Marinobacterium*. In deep sediments, benzoate is oxidized by anaerobic respiratory bacteria (Carmona *et al.*, 2009). Benzoate derived from lignin and crude oils easily accumulates in the upper layer of sediments where oxygen is scarce, yet sunlight is available, and there is a syntrophic interaction between purple sulfur bacteria and sulfur compound-reducing bacteria that contributes to degradation. Tetrathionate is an effective chemical for oxidizing aromatic compounds and tetrathionate-reducing bacteria, such as *Marinobacterium*, which tolerate O_2_ and prefer the upper layer of sediments, in contrast to sulfate-reducing bacteria that are sensitive to O_2_.

Interspecies metabolic interactions have been shown to stimulate growth and metabolic activity in various microbial communities (Zuñiga *et al.*, 2020; Abreu and Datta, 2021; Giri *et al.*, 2021; Abada *et al.*, 2023; Cerruti *et al.*, 2023; Wienhausen *et al.*, 2024). In syntrophic unidirectional interactions, one member scavenges inhibitory metabolites or provides essential nutrients for a partner, *e.g.*, electron sources, respiratory electron acceptors, carbon and nitrogen sources, and growth factors, such as vitamins and amino acids (Mountfort *et al.*, 1984; Schocke and Schink, 1997; Elshahed *et al.*, 2001; Qiu *et al.*, 2003; Summers *et al.*, 2010; Therien *et al.*, 2014; D’Souza *et al.*, 2018; Diender *et al.*, 2021; Gao *et al.*, 2022). In cases where high concentrations of nutrients exert suppressive effects on bacterial growth, the continuous supply of nutrients at low concentrations is necessary for the partner (Pérez *et al.*, 2015; Kawai *et al.*, 2019a). Bidirectional cross-feeding makes interspecies relationships mutualistic and tight (Kawai *et al.*, 2019a; Murakami *et al.*, 2022); a representative example is photoautotrophs providing fixed carbon for a heterotrophic partner and receiving a vitamin from the heterotroph (Kazamia *et al.*, 2012; Xie *et al.*, 2013). The interspecies interactions found in the present study (Fig. 7) involved not only continuous bidirectional cross-feeding (*i.e.*, a carbon source for the autotroph, strain PS1 and a respiratory electron acceptor for the heterotroph, strain BA1), but also scavenging the inhibitory metabolite. These multiple close interactions strengthen their relationships and stimulate metabolic activity.

## Citation

He, M., Nishitani, S., and Haruta, S. (2025) Syntrophic Interaction between an Anoxygenic Photosynthetic Bacterium and a Tetrathionate-reducing Bacterium in Anaerobic Benzoate Degradation. *Microbes Environ ***40**: ME24105.

https://doi.org/10.1264/jsme2.ME24105

## Supplementary Material

Supplementary Material
