# Supplementary material for: Syntrophic Interaction between an Anoxygenic Photosynthetic Bacterium and a Tetrathionate-reducing Bacterium in Anaerobic Benzoate Degradation
Source: Microbes Environ. 2025 Mar 11;40(1):ME24105. doi: 10.1264/jsme2.ME24105 (PMC11946414; doi:10.1264/jsme2.ME24105)
Supplement: Supplementary file 1 — Supplementary Material [file 40_24105_s1.pdf]

Supplemental Materials

Title

Syntrophic interaction between an anoxygenic photosynthetic bacterium and a tetrathionate-reducing bacterium in anaerobic benzoate degradation

Authors

Miao He,[1] Shin-ichi Nishitani, [1] Shin Haruta[1]

Affiliations

[1] Department of Biological Sciences, Tokyo Metropolitan University, 1-1 Minami-Osawa, Hachioji, Tokyo 192-0397, Japan

Correspondence

Shin Haruta, sharuta@tmu.ac.jp

Supplemental Figure S1 Absorption spectrum of the enrichment culture. The enrichment culture was anaerobically cultivated in the benzoate medium to the stationary phase of the growth. Absorption spectrum of the culture solution was determined.

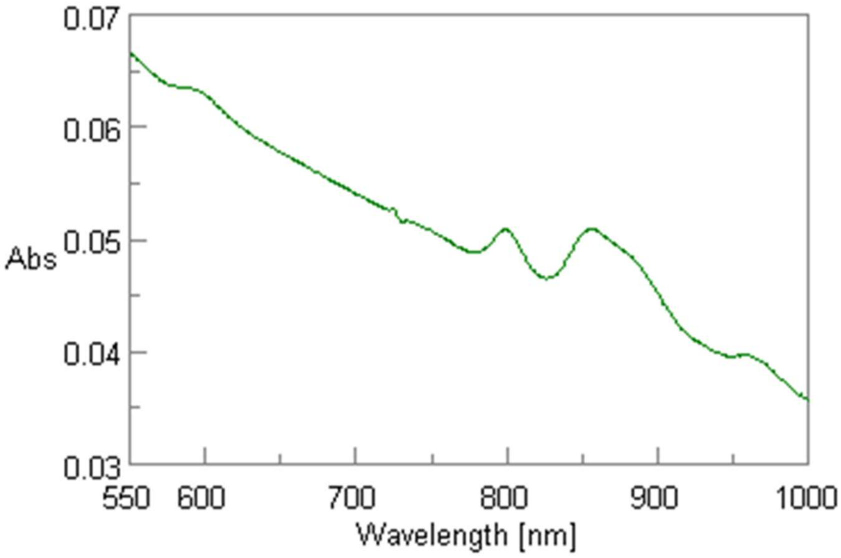

Supplemental Figure S2 Co-cultivation of strain PS1 and strain BA1 in the benzoate medium containing thiosulfate (w/ thiosulfate) and no thiosulfate (w/o thiosulfate) in the light or dark. Anaerobic conditions were achieved by filling the culture tubes with the medium. The optical density (OD) of the culture was measured at 660 nm. Data are shown as the mean of triplicate culture tubes, and error bars show the standard deviation.

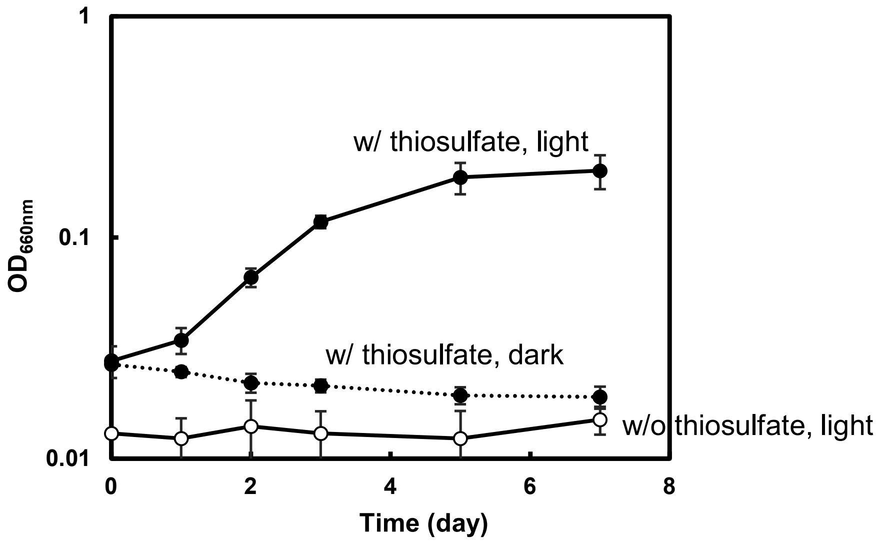

Supplemental Figure S3 Phase-contrast microscopic images for the bacterial cultures. **a**, co-culture of strain PS1 and strain BA1 for nine days in the benzoate-thiosulfate medium as shown in Fig. 3; **b**, axenic culture of strain PS1 for seven days in the benzoate-thiosulfate medium supplemented with $NaHCO_3$ as shown in Fig. 4. Scale bars represent 20 μm. Bottom, close-up view of the area surrounded by a dotted square in each top image.

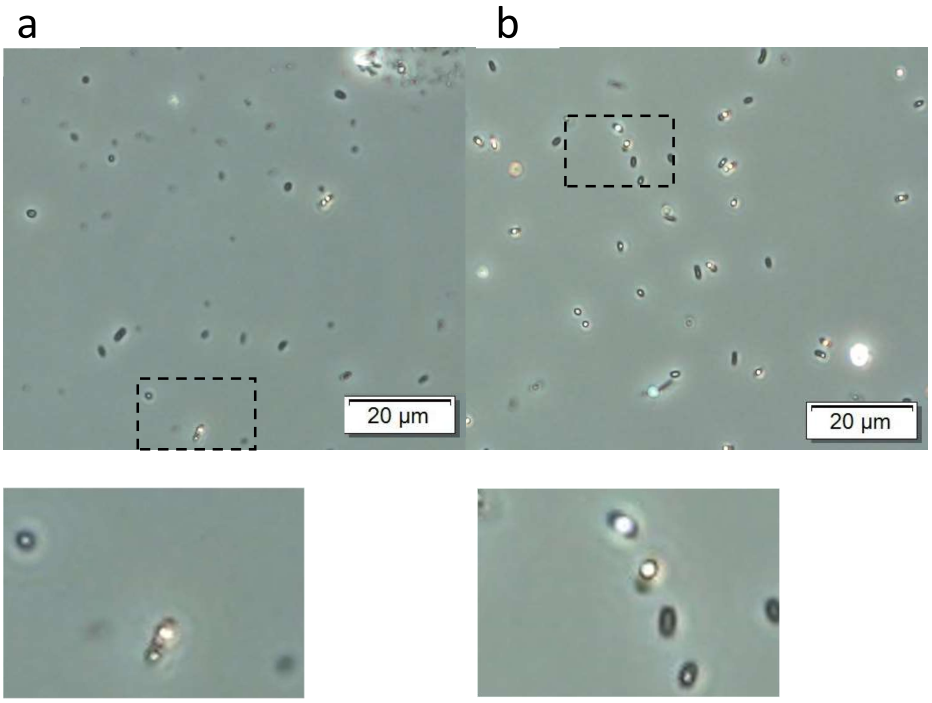

Supplemental Figure S4 Absorption spectrum of the co-culture in the benzoate-tetrathionate medium (as shown in Fig. 6) on day 0 (dashed line) and day 7 (solid line).

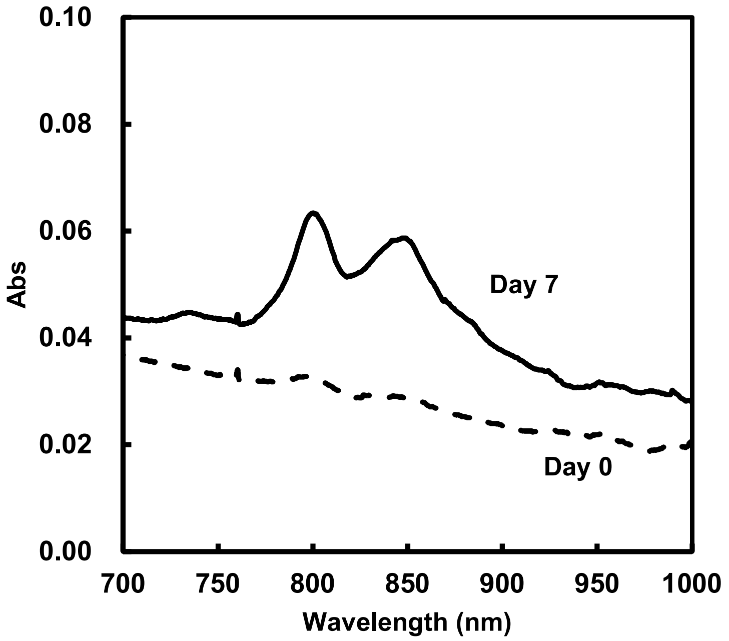

Supplemental Table S1 Summary of genome data of the isolates in this study.

	Marinobacterium sp. strain BA1	*Marichromatium* sp. strain PS1
Accession no.	GCA_041155135	GCA_041155115
Total reads (bp)	248,454,320	247,794,313
N50 (bp)	3,976,056	764,207
Completeness*	100.0%	100.0%
Coverage	58.1	65.0
Contig	2	8
Genome size (bp)	4,273,024	3,812,027
G + C content	55.0%	68.4%
Coding sequences	4,140	3,340
rRNA	15	9
tRNA	72	63

*examined by CheckM2 ver. 1.0.1

Supplemental Table S2 Gene information for benzoate degradation and tetrathionate reduction in the genome of *Marinobacterium* sp. strain BA1.

Gene	Locus tag	Amino acid length	Similarity (%)
Aerobic benzoate degradation			
benzoate hydroxylase			
benA	MnBA_19890	446 aa	99.8%[1]
benB	MnBA_19880	164 aa	99.4%[1]
benC	MnBA_19870	337 aa	99.1%[1]
dihydroxycyclohexadiene carboxylate dehydrogenase			
benD	MnBA_19860	261 aa	99.2%[1]
Anaerobic benzoate degradation (benzoyl-CoA ligase)			
bclA	MnBA_13260	548 aa	44.0%[2]<br>41.9%[3]<br>42.5%[4]
Tetrathionate reduction (tetrathionate reductase)			
tsdA	MnBA_34150	330 aa	43.1%[5]

[1] Similarity with the gene product from the closest relative, *Marinobacterium iners* IC961 (accession number, GCF_017310015.1).

[2] Similarity with the gene product from the anaerobic benzoate-degrading bacterium, *Sedimenticola selenatireducens* BK-1 (accession number, GCF_007625115.1).

[3] Similarity with the gene product from the anaerobic benzoate-degrading bacterium, *Thauera aromatica* K172 (accession number, GCF_003030465.1).

[4] Similarity with the gene product from the anaerobic benzoate-degrading bacterium, *Geobacter metallireducens* GS-15 (accession number, GCF_000012925.1).

[5] Similarity with the gene product from the tetrathionate respiratory bacterium, *Campylobacter jejuni* NCTC 11828 (accession number, GCF_000017905.1).

Supplemental Table S3 Gene information for thiosulfate oxidation and photosynthesis in the genome of *Marichromatium* sp. strain PS1.

Gene	Locus tag	Amino acid length	Similarity (%)*
Thiosulfate oxidation			
tsdBA	McPS_12240	545 aa	99.6%
soxA	McPS_32190	279 aa	98.9%
soxB	McPS_32210	593 aa	99.7%
soxX	McPS_32200	126 aa	100%
soxY	McPS_01070	157 aa	99.4%
soxZ	McPS_01060	104 aa	99.0%
Photosynthesis			
bchZ	McPS_00290	482 aa	99.8%
bchY	McPS_00300	493 aa	99.2%
bchX	McPS_00310	323 aa	96.9%
bchC	McPS_00320	319 aa	100%
pufL	McPS_00260	278 aa	99.6%
pufM	McPS_00250	324 aa	100%
pufC	McPS_00240	355 aa	99.7%

* Similarity with the gene product from the closest relative, *Marichromatium gracile* YL28 (accession number, GCF_001583505.1).
